# Video Game Telemetry as a Critical Tool in the Study of Complex Skill Learning

**DOI:** 10.1371/journal.pone.0075129

**Published:** 2013-09-18

**Authors:** Joseph J. Thompson, Mark R. Blair, Lihan Chen, Andrew J. Henrey

**Affiliations:** 1 Department of Psychology, Simon Fraser University, Burnaby, British Columbia, Canada; 2 Cognitive Science Program, Simon Fraser University, Burnaby, British Columbia, Canada; 3 Department of Statistics, Simon Fraser University, Burnaby, British Columbia, Canada; University of Leuven, Belgium

## Abstract

Cognitive science has long shown interest in expertise, in part because prediction and control of expert development would have immense practical value. Most studies in this area investigate expertise by comparing experts with novices. The reliance on contrastive samples in studies of human expertise only yields deep insight into development where differences are important throughout skill acquisition. This reliance may be pernicious where the predictive importance of variables is not constant across levels of expertise. Before the development of sophisticated machine learning tools for data mining larger samples, and indeed, before such samples were available, it was difficult to test the implicit assumption of static variable importance in expertise development. To investigate if this reliance may have imposed critical restrictions on the understanding of complex skill development, we adopted an alternative method, the online acquisition of telemetry data from a common daily activity for many: video gaming. Using measures of cognitive-motor, attentional, and perceptual processing extracted from game data from 3360 Real-Time Strategy players at 7 different levels of expertise, we identified 12 variables relevant to expertise. We show that the static variable importance assumption is false - the predictive importance of these variables shifted as the levels of expertise increased - and, at least in our dataset, that a contrastive approach would have been misleading. The finding that variable importance is not static across levels of expertise suggests that large, diverse datasets of sustained cognitive-motor performance are crucial for an understanding of expertise in real-world contexts. We also identify plausible cognitive markers of expertise.

## Introduction

Work in expertise and skill learning most often follows one of two paradigms: making precise measurements of performance, but with poorly trained participants doing relatively simple laboratory tasks [1], [2], [3], [4] or studying real-world experts while taking only indirect measures of domain performance [5] from two or three levels of skill [6], [7], [8], [9]. The applicability of these paradigms to understanding the development of expertise rests on the validity of extrapolating from short-term laboratory training or from interpolating from long-term comparisons between experts and novices. These methodologies are thus highly informative where skill development is a smooth transition between expert and novice, but may be problematic if the skill level of the participants changes whether or not a process is important to success. For example, the method is deeply problematic in the comparison of 10 month old infants and 20 year old college students. The two groups could obviously be distinguished by the capacity to pass traditional false belief tasks and by the capacity for algebra, but it does not follow that false belief tests are useful for distinguishing 15 and 20 year olds, or that such tests are even relevant to studying this period of development. Similarly, contrastive methods in the study of expertise are potentially misleading if variable importance changes throughout development. Given that expertise encompasses years of training and significant cognitive motor change, the assumption that variable importance remains static warrants investigation. There is some evidence in the motor learning literature that variable importance can change over small amounts of training (<10 hours) in relatively simple laboratory tasks [4]. Whether changes in variable importance exist on the longer timescale of the development of expertise, especially expertise involving a substantial cognitive component, is unclear. One possible source of evidence could be found in medical expertise, as some authors report that the relationship between expertise and the number of propositions recalled from a medical diagnosis follows an inverted-U shaped function [10] implying that the utility of this predictor varies depending on the levels of expertise being compared. The variable may, for example, be less useful for distinguishing novices and experts than it is for distinguishing intermediates and experts. Until recently, however, there was no straightforward and direct way to test the assumption of static variable importance in a *rich*, *dynamic*, *realistic* context.

Here we use the analysis of video game telemetry data from real-time strategy (RTS) games to explore the development of expertise. Expertise in strategy games has long been a subject of interest for researchers [9], [11], [12], [13]. This is not because there is some expectation that expert chess players will be more savvy generals, or that expert tennis players are likely to be better pilots. While the knowledge and skills do not transfer, there is enough consistency in the development of expertise that unified theories have been developed [14]. One would therefore expect that the development of RTS expertise would resemble the development of expertise in these and even less related domains, such as surgery.

RTS games, in which players develop game pieces called units with the ultimate goal to destroy their opponent’s headquarters, have three relevant differences from traditional strategy games such as chess. First, the games have an economic component such that players must spend resources to produce military units. Many of a player’s strategic decisions are related to balancing spending on military and economic strength. Second, the game board, called a map, is much larger than what that player can see at any one time. The resulting uncertainty about the game state leads to a variety of information gathering strategies, and requires vigilance and highly developed attentional processes. Third, in RTS games players do not have to wait for their opponent to play their turn. Players that can execute strategic goals more efficiently have an enormous advantage. Consequently, motor skills with a keyboard and mouse are an integral component of the game. Each game produces lots of behavioral data: an average game of chess consists of 40 moves [15] per player, while the average RTS game in our study consists of 1635 moves per player.

We bear the burden of arguing that RTS play can be considered an area of expertise in the same sense that chess or Go are areas of expertise. Playing well requires a great deal of strategy and knowledge, and these require a great deal of experience. It satisfies the definition of expertise as being “characteristics, skills and knowledge that separates experts from novices and less experienced people” (p. 3) [16]. Skilled StarCraft players perform consistently better than less skilled ones, as evidenced by the game developer’s need to develop a matchmaking system for fair play. RTS games also meet more commonplace notions of expertise (such as athletic expertise) grounded in professional performance requiring skills and commitment far beyond that of average individuals. StarCraft 2 supports a variety of professional and semi-professional players. Top players can earn 250,000 USD a year [17], motivating full time commitment to the game. Professional’s practice 6–9 hours a day, 6 days a week and often have a decade or more of RTS experience. Tournaments are broadcast live and professional teams are sponsored by major corporations. All of this is evidence that RTS gaming is a domain of expertise. Furthermore, competence in the game necessarily involves fast and meaningful hand movements and intelligent control of the game’s view-screen in order to see and act, so it follows that attention, perception, decision making, and motor control (all of which we will collapse under the term “cognitive-motor abilities”) are important to StarCraft 2 expertise.

By studying a domain of expert performance that is entirely computer-based, we are able to obtain accurate measures of performance in its natural environment By using an existing, popular and competitively played video game we are able to obtain much larger diverse samples via online correspondence from participants all over the world. The present study analyzes data from 3,360 StarCraft 2 players across 7 distinct levels of skill called leagues (Bronze, Silver, Gold, Platinum, Diamond, Masters, Professional), making it the largest expertise study ever conducted. Our research goal was to identify potential markers of expertise in RTS games (with special interest in general cognitive markers), and to form a clear picture of the complexity of expertise.

When these aspects of RTS games are taken in conjunction with the telemetric collection and analysis of detailed game records, we are left with a project that has the following virtues:

1. A rich, dynamic task environment,
2. Highly motivated participants,
3. Accurate measures of motor performance and attentional allocation,
4. Noninvasive and direct measures of domain performance,
5. Large datasets,
6. Numerous variables,
7. Many levels of expertise.

This approach is therefore uniquely situated for exploring expert development.

## Materials and Methods

### Ethics Statement

This study was reviewed and approved by the Office of Research Ethics at Simon Fraser University (Study Number: 2011s0302). Participants provided informed consent in an online survey.

### Data Collection

Telemetric data was collected from 3,360 RTS game players from 7 levels of expertise, ranging from novices to full-time professionals. We posted a call for StarCraft 2 players through online gaming communities and social media. From each respondent we gathered a replay file (a recording of all the commands issued in the game), demographic information, and a player identification code that allowed us to verify their level of expertise (as measured by the league in which they compete - online competitive leagues are comprised such that 20% of players are Bronze, 20% Silver, 20% Gold, 20% Platinum, 18% Diamond, and 2% Masters [18]). Replays of professional players’ games were obtained from gaming websites.

The primary research question does not depend on any particular variable but on the pattern of importance of all the variables across the levels of expertise. Nevertheless, we selected predictor variables that relate to cognitive-motor abilities. In addition, we selected variables that relate to cognitive load, that is, the amount of mental energy required to perform the task. Unlike laboratory tasks, many of which ask participants to do a single simple task, success at StarCraft2 requires the completion of many separate but interrelated tasks. This can lead to difficulties, as there are serious constraints on attention which limits the ability to perform multiple tasks concurrently [19]. There is much work on skill learning which indicates that after extensive practice, people not only perform tasks more quickly and accurately, but they also to require fewer cognitive resources to perform and become nearly effortless. This is typically called automaticity (e.g., Logan [2], Schneider & Shiffrin [20], Shiffrin & Schneider [21]) in cognitive psychology. Related concepts can be found in other fields. For example, one concern of research on Unmanned Ground Vehicle operation is how the degree to which navigation is autonomously controlled by computer systems affects operator mental workload [22]. The variables chosen for the present study, which reflect the considerations above, fall under the following categories:

Perception-Action-Cycle variables. Each variable pertains to a period of time where players are fixating and acting at a particular location. Many of these variables will therefore reflect both attentional processes (because Perception-Action-Cycles have consequences for what players are able to attend to), perceptual processes (because shifts of the screen imply new stimuli), and cognitive-motor speed (in the sense that actions must not only be fast but meaningful and useful).

Hotkey usage variables. Players can customize the interface to select and control their units or building more rapidly, thus offloading some aspects of manually clicking on specific units to the game interface.

Complex unit production and use variables. Certain units pose dual task challenges and some need to be given explicit direction or targeting instructions. The production and use of these units and abilities is sometimes optional, and so their production and use may reflect a player’s modulation of their own cognitive load.

Direct measures of attentional control. StarCraft 2 presents a number of attentional challenges for players. One challenge is that the primary view screen contains detailed and highly salient information that potentially distracts players from the less detailed information of the entire map (this “mini-map” occupies a small portion on the bottom-left of their screen). Mini-map variables reflect player’s performing of actions on the mini-map, and we hypothesized that better players would do a better job of attending to, and using, this map. We also considered how much of the total map was looked at by players, which we thought relevant to the seeking of information about the game state.

Actions per minute. This variable is often used as a predictor of expertise in the StarCraft community and is automatically calculated by the game. It is a measure of cognitive motor speed.

The Supplementary Materials (Materials S1) contain complete definitions for all variables in the analysis.

We extracted a list of all the actions and screen moves from each game replay file. Players move their screen to different locations on the map to perform actions at those locations or to gather information about what is occurring at those locations. These screen moves are very like saccadic eye-movements. To deal with this problem we aggregated screen movements into PoVs using the fixation-IDT algorithm in Salvucci & Goldberg [23], with a dispersion threshold of 6 game coordinates and a duration threshold of 20 Timestamps (about 230 milliseconds). The algorithm aggregates screen movements to provide (a) a pair of Cartesian screen coordinates and (b) a duration for each PoV. Proceeding from earliest screen movements to later ones, the algorithm first collects the smallest set of screen movements such that adding another screen movement would exceed the duration threshold. If the dispersion (defined as [(max(x) - min(x))+(max(y) - min(y))]) of these points exceeds the dispersion threshold, the first screen movement is dropped from the set of screen movements and the process is repeated. If the dispersion of the set does not exceed the dispersion threshold, then a new screen movement is added to the set and the process is repeated until adding a new screen movement produces a window that fails to satisfy the dispersion threshold. The coordinates of screen movements in the set are then averaged into PoV coordinates and the PoV is said to begin at the earliest screen movement in the set and end at the point where the dispersion threshold is violated.

The definition of PoVs allowed the analysis of PoVs that contain one or more actions, which we call Perception Action Cycles (PACs). Hotkey selects are not considered an action for calculating any PAC variable as these actions may also be used to produce new PoVs themselves. PACs encompass roughly 87% of the participants’ game time. This finding echoes research using eye-tracking to record gaze while participants do real world tasks [24]. This work found that participants’ PoVs are predominantly part of sequences of object related actions. PACs also make a useful parallel to individual trials within a laboratory experiment in which the participant perceives stimuli and makes a series of responses. For example, Action Latency, the time from the onset of a PoV to the first action, is a close analogue to reaction time in laboratory experiments.

In order to ensure comparability between games, we restricted our analysis to rated competitive ladder games between two humans that lasted longer than five minutes, were played at the same game speed, and were played on a StarCraft 2 versions 1.3.6.19269∶291 (also see exclusion criteria). This ensures that each game had essentially the same starting conditions. Two important exceptions are that games are sometimes played on different maps, and that players may occupy different starting positions (although competitive ladder maps are typically symmetrical and are balanced to ensure fair games).

### Exclusion Criteria and Sample Characteristics

Of the 9222 Participants who began the process of filling out the survey, 5917 were dropped from the study for satisfying one of the following exclusion criteria:

1. Participant failed to supply a Battle.net ID for league verification: 4706
2. Participant failed to submit a valid replay file: 191
3. Game had a Max Timestamp smaller than 25000 (roughly 5 minutes): 72
4. Game was played with more or less than two human players: 44
5. Game was played at a game-speed slower than “faster”: 5
6. Participant did not have a 1v1 ranking on Battle.net: 141
7. Game was not a grandmaster game, and was played in leagues that were not played using Blizzard’s “Automatchup” feature: 356
8. Game had fewer than 100 commands and screen movements overall: 0
9. Game was not a professional game, and was played on a version of StarCraft 2 other than 1.3.6.19269∶291
10. Participant submitted a Battle.net ID, but it did not match any player in the game: 76
11. The game was not a 1 versus 1 game: 0
12. Belonged to the league Grandmasters: 35

The survey data includes 7 leagues (Bronze, Silver, Gold, Platinum, Diamond, Masters, Grandmasters). The sample of Grandmasters participants was significantly smaller than that of the other leagues. This was not surprising as the Grandmasters league in Starcraft 2 included only the top 200 players in each region – a population smaller in orders of magnitude than that of the other leagues. These 200 players consisted of both top casual players and professional players, which could not be distinguished independently of the variables used in the analysis. Due to the analytic difficulties of this group imposed, we dropped the data from the analysis. Instead, we were able to obtain a larger and more homogenous group, the professionals, from 55 additional publicly available games collected online from professional StarCraft 2 players who competed in the GomTV StarCraft League (GSL) tournament (the most prestigious tournament in competitive StarCraft) in July 2011 or August 2011.

The sample size by league was as follows:

Bronze : 167.

Silver: 347.

Gold: 553.

Platinum: 811.

Diamond: 806.

Masters: 621.

Professional: 55.

Participants reported their countries of origins. According to the survey results, participants came from 77 countries, primarily the United States (1425), Canada (480), Germany (246), and the United Kingdom (187). Participants’ ages ranged from 16–44 (Median = 21; Mean = 21.6; SD = 4.2), which included 3276 males and 29 females. The one-tail 95% trimmed mean of reported hours of Starcraft 2 experience was 545, and the mean of reported StarCraft 1 experience was 4.07 years. Histograms for each variable (by league) are given in Supplementary Figures S1–15.

### Analysis

The primary theoretical question is whether the predictive importance of variables is stable across levels of experience. To answer this, we evaluated variable importance across skill levels by creating a series of statistical classifiers that distinguished players from two different leagues. An important challenge we encountered with this dataset is that the players are grouped into somewhat heterogeneous skill classes. The placement of players into leagues does not perfectly reflect skill, and a high-ranking player within a class might be objectively a better player than a lower-ranked player in the class above. As a consequence, the classes directly beside each other are not separable, and we found that classifiers performed poorly when trying to distinguish between neighboring classes. We had significantly more success when we used the variables to predict class when the distance between classes was at least two. Our method of determining variable importance across skill thus consists of a series of two-league classifiers, each based on classes two leagues apart (e.g. Bronze-Gold). We include a final classifier that emulates the contrastive (novice-expert) approach by comparing only the most extreme skill levels (Bronze and Professional).

Although logistic regression is an option for two-class classification, we preferred to use the more flexible conditional inference forest algorithm, which has emerged from work on random forest classifiers [25], [26], [27] (for more information see the work of Carolin Strobl [28]). The main advantage of using conditional inference forests over logistic regression is that we do not need to make unnecessary assumptions about the structure of the relationship between the predictive variables and the response. Furthermore, these classifiers do not exhibit some of the biases present in other random forest techniques [29]. However, random forests in general do not come with significance tests, so we needed to adopt a suitable procedure, which is discussed below.

The forests were created using the cforest function in R with ntree = 1000 trees and mtry = 5 variables per split. We assess the randomness in the algorithm by running the forest on samples of size 70% drawn without replacement from the original data twenty five times, as a distribution of importance scores is required by the decision procedure in Linkletter et al. [30]. The Conditional Inference Forest algorithm gives a measure of variable importance called permutation importance index for each variable, but it does not give a p-value for a hypothesis test against zero (see supplementary methods and materials in Materials S1). We follow Linkletter et al. by adding our own random noise variable as a control variable each time we subsample the data, and the 95th percentile of this distribution serves as a critical value for a test against the null [30]. It is important to note that this method does not control for a particular family-wise type 1 error. This is reasonable for screening research such as ours [30], where the goal is to identify variables worthy of further research. Our research sets the stage for further studies that will confirm the importance of variables identified here and probe the relations between them.

## Results

**Figure 1:**
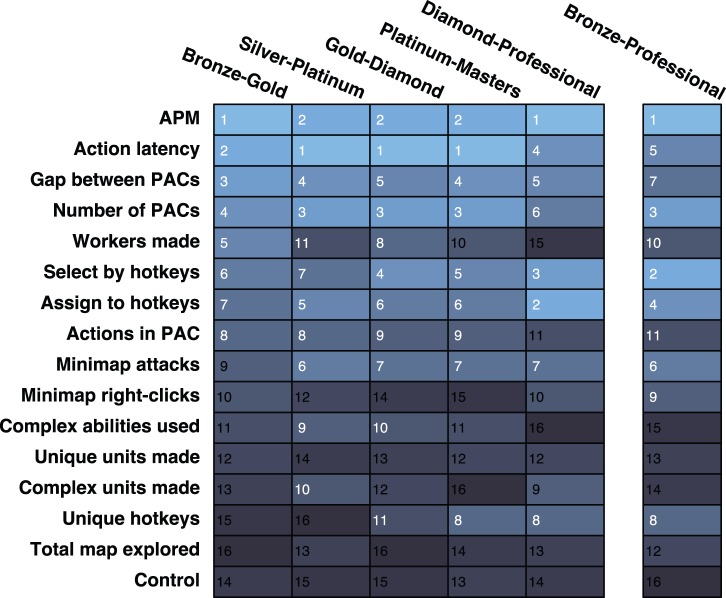
Variable Importance. The rank of the permutation importance for each variable. Each column refers to a random forrest classification. Grid colors and numbers reflect ranks. White numbers indicate that the variable is above the cuttoff defined by the control variable (see Supplementary Materials and methods in Materials S1).

**Figure 2:**
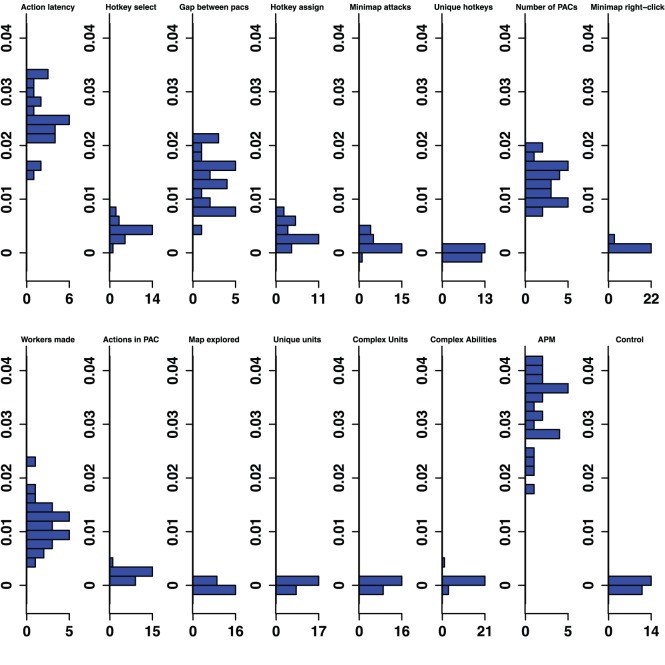
Bronze-Gold Permutation Importance. Histograms for permuted importance values from 25 conditional inference forests for each of the 16 variables used in the Bronze-Gold classifier. Classification rate: 82.32%. Baseline accuracy of classifier from choosing more common class: 77.81%.

**Figure 3:**
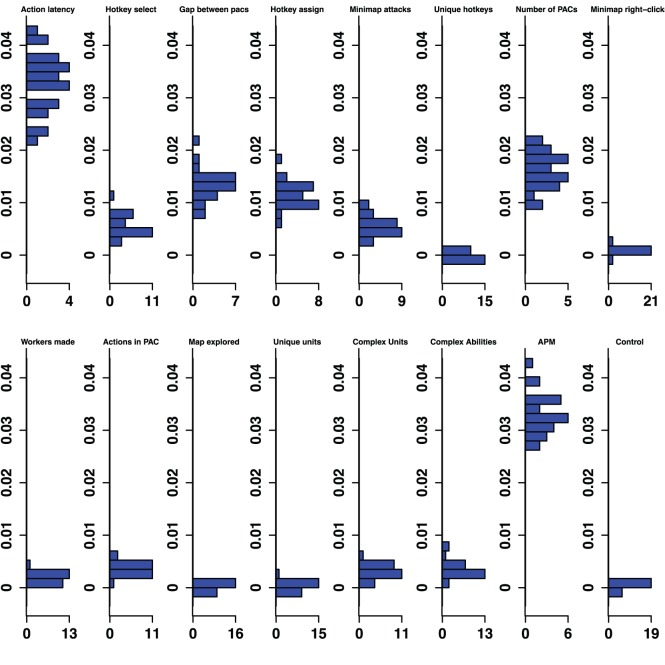
Silver-Platinum Permutation Importance. Histograms for permuted importance values from 25 conditional inference forests for each of the 16 variables used in the Silver-Platinum classifier. Classification rate: 78.27%. Baseline accuracy of classifier from choosing more common class: 70.03%.

**Figure 4:**
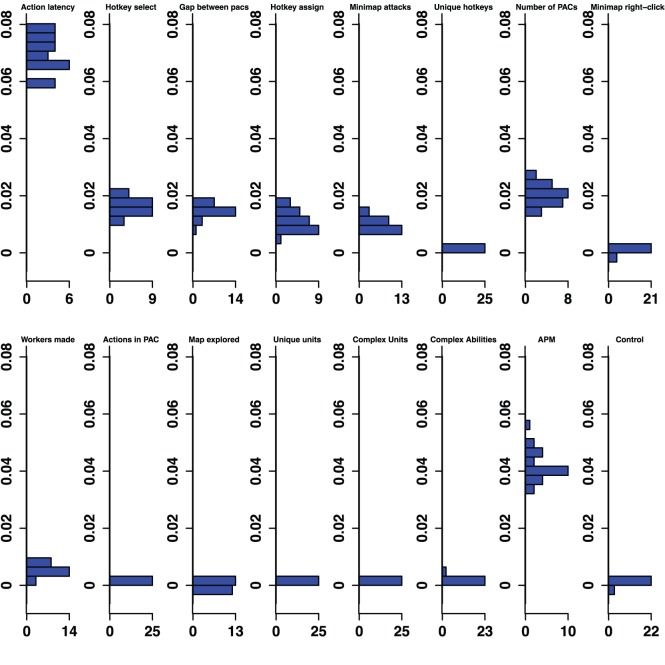
Gold-Diamond Permutation Importance. Histograms for permuted importance values from 25 conditional inference forests for each of the 16 variables used in the Gold-Diamond classifier. Classification rate: 79.01%. Baseline accuracy of classifier from choosing more common class: 59.31%.

**Figure 5:**
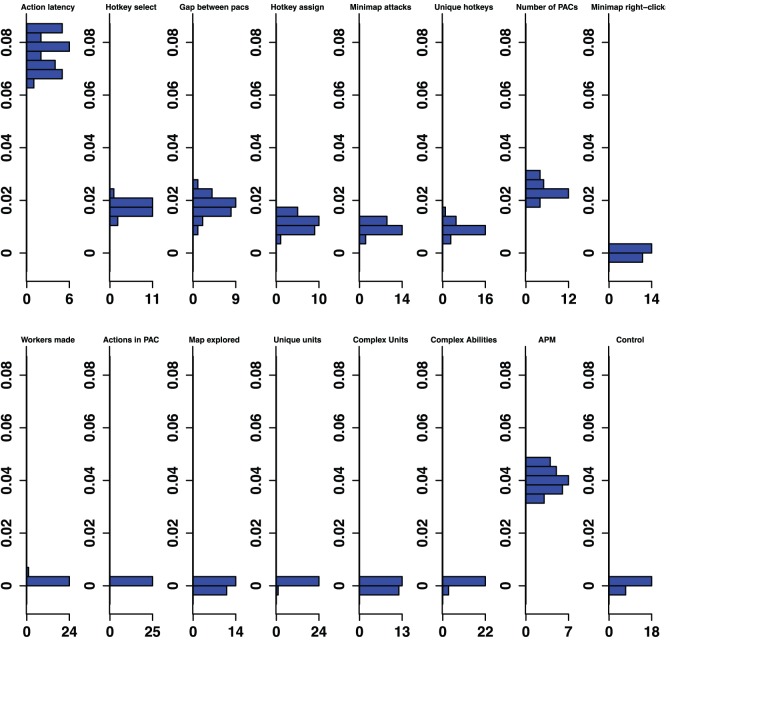
Platinum-Masters Permutation Importance. Histograms for permuted importance values from 25 conditional inference forests for each of the 16 variables used in the Platinum-Master classifier. Classification rate: 80.70%. Baseline accuracy of classifier from choosing more common class: 56.63%.

**Figure 6:**
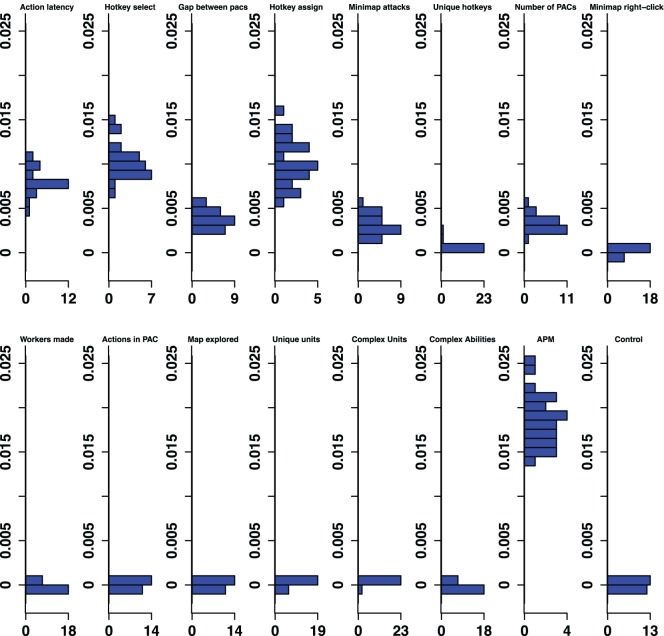
Diamond-Professional Permutation Importance. Histograms for permuted importance values from 25 conditional inference forests for each of the 16 variables used in the Diamond-Professional classifier. Classification rate: 96.75%. Baseline accuracy of classifier from choosing more common class: 93.61%.

The classifier analysis provided a method of ranking the importance of variables at multiple levels of skill. Figure 1 provides the rank of the predictive importance for the 15 variables in each classifier, along with the control variable. For example, Action Latency had the second highest median permutation importance in the Bronze-Gold classifier, and had the highest median permutation importance in distinguishing Silver-Platinum classifier. Figures 2–6 show histograms of the permutation importance index of the variables along with the control value for the 25 samples of the data, and reveal the details of the importance index for each classifier used to determine the ranks shown in Figure 1. Variables which proved more important than the control variable, that is, their median importance index (shown in Figures 2–6) was outside the range of the importance index found for the control (probability <5%), have rank importance values in Figure 1 shown in white. Rank values shown in black, were not more important than control. Overall, twelve variables were useful predictors in at least one of the five league-specific classifiers (shown in Figure 1), but only six were predictive in all five classifiers. This result supports the hypothesis that variable importance is not stable across expertise.

Furthermore, even variables that were important in all five classifiers changed in rank importance. Action Latency in particular was one of the two strongest predictors of expertise in four of the five classifiers, and was ranked fourth in the Diamond-Professional classifier. The importance of PAC variables shifts across classifiers. Action Latency was unequivocally the most important variable in the Gold-Diamond and Platinum-Masters classifiers, where its distribution of permutation importance values did not overlap with those of any other variable (see Figures 4 and 5). However, the situation was markedly different in the Diamond-Professional classifier, where Actions Per Minute was more important than Action Latency in all 25 runs of the classifier (Figure 6). Like PAC variables, other variables also changed in importance. This demonstrated that variables do indeed change in importance across the skill continuum.

Because there was unequal class size and unequal numbers of data points used to build the different classifiers, there was some concern that the finding of changing variable importance was an artifact. To investigate this possibility we reran the classifiers with 125 players per class (we excluded the professional games, as we had only 55 professional games). The main findings are essentially unchanged: we still observe variable importance changing across classifiers, although some of the detail is lost, due to the significant reduction of data. Rank variable importance shifts somewhat, which is to be expected because of the randomness in the process of building the classifiers. Nevertheless, the main predictor variables still beat the control variable, and “Workers Trained” remained important in lower league classifiers but did not beat the control in higher leagues. After rerunning the analysis with equal class sizes, and again with equal classifier sizes, we find no reason to believe the results of the original analysis are artifacts.

**Figure 7:**
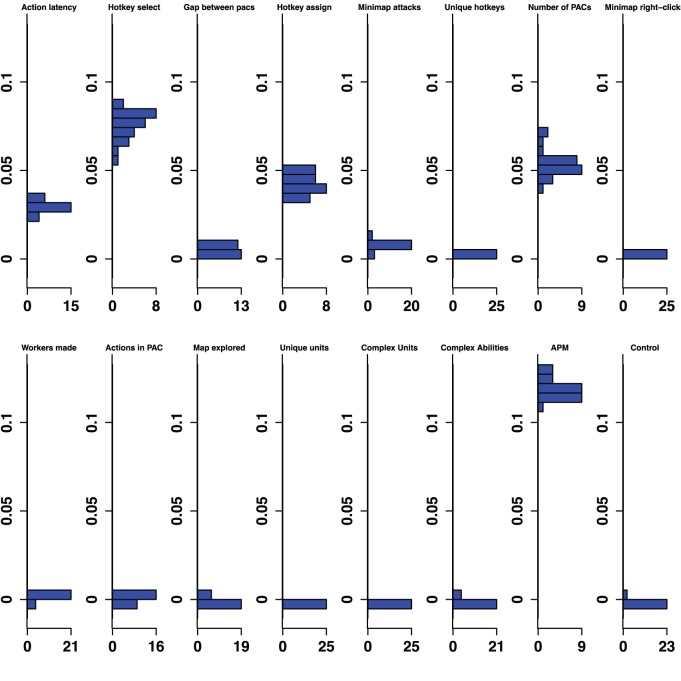
Bronze-Professional Permutation Importance. Histograms for permuted importance values from 25 conditional inference forests for each of the 16 variables used in the Bronze-Professional classifier. Classification rate: 98.59%. Baseline accuracy of classifier from choosing more common class: 75.23%.

The contrastive approach, which mimics the expert/novice comparisons used in so many studies of expertise, is emulated by a classifier which separates players in the lowest league from professionals, the Bronze-Professional Classifier shown on the right column of Figure 1. If our study had used this approach, it would have clearly missed important features of development. Furthermore, the results it would have produced would also be misleading. For example, the Bronze-Professional classifier would have overestimated the importance of Hotkey Selects relative to Action Latency. In fact, Hotkey Selects were excellent in distinguishing Bronze and Professional players, where it was the second most important predictor (Figure 1), and had a permutation importance distribution that was higher, and did not overlap with, the importance distribution of Action Latency (Figure 7). However, Hotkey Selects never enjoyed this kind of clear importance over Action Latency in any of the other classifiers. On the contrary, Action Latency was more important than Hotkey Selects in all 25 runs of the Bronze-Gold, Silver-Platinum, Gold-Diamond, and Platinum-Masters classifiers (Figures 2–5). The contrastive approach could have lead researchers away from what appears to be a very important measure of cognitive-motor performance.

**Figure 8:**
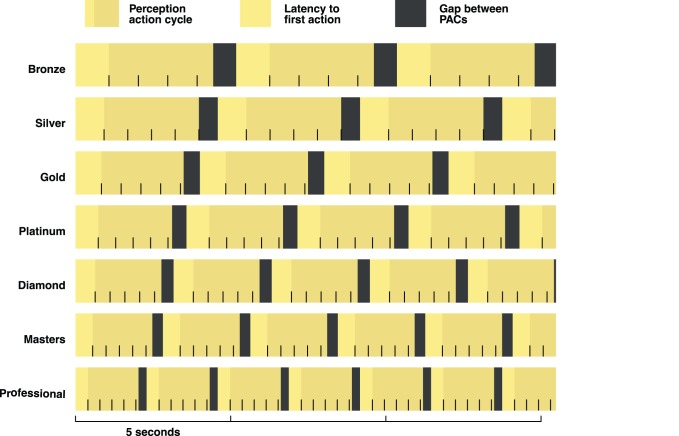
Perception Action Cycles (PACs). Actions and attention shifts for a typical StarCraft 2 player over 15 seconds. Each vertical line tic represents a single action. Notice that most aspects of the PAC become faster with an increase in League.

Beyond the primary theoretical finding, we discovered unequivocal evidence for the general importance of all PAC variables as cognitive markers of expertise. As Perception-Action-Cycles partition behavior into looking-doing couplets, PAC variables might capture a host of interesting cognitive-motor variables. Perhaps the most interesting of these is Action Latency, which likely reflects perceptual and decision making processes. Figure 8 shows the typical PAC compression from Bronze League to Pro, and provides an overview of the PAC variables. Additional line tics indicate the typical number of actions within a PAC. For example, Bronze players typically take five seconds to complete a PAC and move to the next PAC, and the mean number of actions within each Bronze PAC is a little more than four. Professional players take about half this time. The finding that the number of actions within each PAC remain relatively stable throughout expertise, in particular, is a result worthy of future inquiry.

The overall pattern of changes in variable importance is consistent with the thesis that automatization of some skills, in the sense that tasks can be performed quickly and without intentional control, reduces cognitive load and allows for the development of other skills. For example, the most volatile variable was a marker of economy development, the production rate of workers. This variable was important in lower league classifiers, but not for distinguishing Diamond and Professional players. Continuous worker production is a critical component of success. To succeed, players must constantly switch between worker production and the control of military units, much like in a dual-task study. By Diamond League, players seem to have automatized worker production, relieving them of some cognitive demands. Furthermore, the players’ efforts to manage their cognitive limitations and cope with the increasing cognitive demand as they advance in league may be reflected in the use of hotkeys. The number of Unique Hotkeys used was predictive in the higher leagues and the frequency of Hotkey Selects (used to access previously assigned units) and Hotkey Assignments started out as respectable predictors (ranked 6th and 7th in the Bronze-Gold classifier; Figure 2) and were very strong predictors in the Diamond-Professional classifier (ranked third and second respectively, and even ranked above the PAC variables; Figure 6). Another way that players could modulate the game’s cognitive-motor demands is by employing specific strategies that reduce cognitive load. For example, players in the lowest leagues seem to avoid units and abilities requiring delicate targeting instructions, as reflected in the predictive importance of Complex Ability Use (which was predictive in Silver-Platinum and Gold-Diamond classifiers; Figures 3 and 4) and Complex Units Made (which was predictive in the Silver-Platinum classifier; Figure 3). For each variable used, histograms by league are available in the supplementary materials (Figures S1–15).

## Discussion

The primary finding is that predictors of expertise change in their importance across skill levels. We also demonstrated that a purely contrastive approach produces a distorted view of changes across expertise. These results make the interpretation of contrastive studies and the generalization from laboratory designs more problematic. They also show that the telemetric collection of data can confer deep benefits to the study of skill development.

The results also show that RTS game replays in particular can track abilities of interest to cognitive science. The extreme compression of these cognitive motor measures, the comparative ease of worker production in mid-to higher-skill players, and the increasing importance of using hotkeys are in keeping with the view that automaticity is an important component of expertise development. As some skills are automatized, it frees up cognitive resources for players to devote to learning other skills. Interestingly, this change would also have a profound impact on the learning environment and therefore shape future change. It is important to note, however, that transitions between skill levels may not reflect the process of automaticity alone. Ericsson, for example, argues that conscious control and management of learning are required for individuals to continue to improve particular skills [31]. In our sample, the use of hotkeys are especially pronounced in professional players (see Figure S4,S6), and while this could be because using hotkeys requires substantially more experience than is available to non-professional players, it also may reflect consciously controlled training on behalf of professionals.

The present work has several important limitations. First, our measure of skill, though more fine-grained than typical contrastive studies of expertise, is nonetheless ordinal. This hampers our ability to describe development in a continuous fashion. Given that professionals train many hours a day, it would be helpful, for example, to chart the substantial development from Masters to Professional. The present design also fails to capture expertise changes at the individual level. The average Bronze player has 200 hours of experience, but there is no way to know if a particular player, given another 800 hours of practice will end up in Masters league. Another limitation is that we have only a single game from each participant, thus have no good estimate of the variability of individual performance. We also cannot say anything about individual difference in learning trajectories or whether there multiple pathways to expertise. Finally, the present study is observational, and not experimental, and so causal relationships are not identifiable. Future work is needed, for example, to demonstrate that the number of workers created is automatized by showing that in higher leagues it is less prone to disruption by an additional cognitive load.

While the above limitations apply to the present study, they are not limitations of the general paradigm of analyzing telemetric data from RTS games. Continuous measures of skill exist. In any competitive games, developers need to match players of similar skill, to ensure the games are fair. This is often a continuous measure called their match-making rating. While these data are not always available to researchers, game developers are, at least in our experience, supportive of research efforts. Perhaps more importantly, the method can be adapted to longitudinal designs. Replay files are compact, meaning that many players have accumulated a record of literally every StarCraft 2 game they have ever played. This allows for the sampling of entire ontogenies of expert development in longitudinal studies of human performance on the microgenetic scale. Scientists can also test specific causal hypotheses in RTS games using existing game-modification tools. With StarCraft2, for example, the company includes tools which allow the modification of almost any aspect of the game. The modified games can be published online for other players to use. A massive sample of participants, randomly assigned to conditions by the modified game, can thus be collected telemetrically. The kind of manipulations used to understand chess expertise, for example, are easily implemented, but with larger and more diverse datasets. This was the dream of “Space Fortress”, a game designed by Mané and Donchin [32] to study cognitive-motor development. They wrote: “The goals were (1) to create a complex task that is representative of real-life tasks, (2) to incorporate dimensions of difficulty that are of interest based on existing research on skill and its acquisition, and (3) to keep the task interesting and challenging for the subjects during extended practice” (p. 17). Space Fortress studies have used up to 40 hours of training [33], and this is far more than most skill learning experiments. While this is admirable, the present method can do better. The least skilled group in our study, the Bronze players, report 200 hours of experience on average. Lewis, Trinh, and Kirsh [34] demonstrated that researchers could analyze telemetric data from video games, like StarCraft, that are already extremely popular. Our study develops this paradigm further and motivates additional research into StarCraft 2, which has millions of players worldwide, and allows for easy telemetric data collection, skill verification, and even experimentation. Of course, the research opportunities extend beyond StarCraft 2, as the features making these virtues possible are becoming more common in video games generally.

We have argued that the present paradigm has tremendous advantages on its own, but it can also be used to guide researchers using other methods. For example, if one were interested in studying neural changes involved in multitasking, our data suggest that at least one of the multitasking challenges of StarCraft 2 is overcome in the early leagues (see workers trained per minute, Figure S10). Given the difficulty of acquiring professional players, and the expense of neuroimaging studies, knowing when these skills develop allows researchers to efficiently target specific changes of interest. In this way analysis of telemetric data can provide a kind of map of skill development that can serve as a guide for a variety of research tools and paradigms.

In light of the improvement this method provides over the typical contrastive methods, we propose that RTS games can serve cognitive science as a ‘standard task environment’ [35], as drosophila have served biology. As the number of domains of expertise that are predominantly computer mediated increases, so will the relevance of telemetric data to the study of complex learning. As human computer interactions involve more sensors to record human behavior (such as eye-tracking and biometrics) more interesting real-world performance can be recorded and leveraged to make significant advances in our understanding of human cognition and learning.

## Supporting Information

Figure S1 **Histogram of PAC Action Latency (ms)(M = 719.94, SD = 217.3).**
(EPS)

Figure S2 **Histogram of the Number of actions in each PAC (M = 5.27, SD = 1.49).**
(EPS)

Figure S3 **Histogram of the Number of PACs every minute (M = 18.4, SD = 5.27).**
(EPS)

Figure S4 **Histogram of Select by Hotkeys per minute (M = 22.83, SD = 28.07).**
(EPS)

Figure S5 **Histogram of Gaps between PACs (M = .46, SD = .2).**
(EPS)

Figure S6 **Histogram of Assign by hotkeys per minute (M = 1.99, SD = 1.19).**
(EPS)

Figure S7 **Histogram of Minimap attacks per minute (M = 0.52, SD = 0.88).**
(EPS)

Figure S8 **Histogram of Unique hotkeys (M = 4.36, SD = 2.36).**
(EPS)

Figure S9 **Histogram of Minimap right-clicks per minute (M = 2.06, SD = 2).**
(EPS)

Figure S10 **Histogram of Workers created per minute (M = 5.48, SD = 2.76).**
(EPS)

Figure S11 **Histogram of Map explored (M = 22.13, SD = 7.43).**
(EPS)

Figure S12 **Histogram of Unique units made (M = 6.53, SD = 1.86).**
(EPS)

Figure S13 **Histogram of Complex units made per minute (M = 0.32, SD = 0.59).**
(EPS)

Figure S14 **Histogram of Complex abilities used per minute (M = 0.75, SD = 1.4).**
(EPS)

Figure S15 **Histogram of APM (M = 117.05, SD = 51.95).**
(EPS)

Figure S16 **A simple classification tree.**
(EPS)

Materials S1 **Supplemental Materials and Methods.**
(DOC)

## References

[1] Proctor RW, Vu KP (2006) Laboratory studies of training, skill acquisition, and retention of performance. In the Cambridge handbook of expertise and expert performance, K.A. Ericsson, Charness N, Feltovich PJ, Hoffman RR Eds. Cambridge, MA: CUP. 265–286.

[2] Logan GD (1988) Toward an instance theory of automatization. Psychol. Rev. 95 (4): 492–527.

[3] Palmeri TJ (1997) Exemplar similarity and the development of automaticity. JEP:LMC 23 (2): 324–354. 10.1037//0278-7393.23.2.324 9080007

[4] Fleishman EA, Parker JF (1960) Ability Factors and Component Performance Measures as Predictors of Complex Tracking Behavior. Psychol. Monogr. 74 (16): 1–36.

[5] Charness N, Tuffiash M, Krampe R, Reingold E, Vasyukova E (2005) The Role of deliberate practice in chess expertise. Appl. Cognit. Psychol. 19: 151–165.

[6] Ericsson KA, Williams AM (2007) Capturing naturally occurring superior performance in the laboratory: Translational research on expert performance. JEP:A 13(3): 115–123. 10.1037/1076-898X.13.3.115 17924797

[7] Abreu AM, Macaluso E, Azevedo RT, Cesari P, Urgesi C, et al (2012) Action anticipation beyond the action observation network: a functional magnetic resonance imaging study in expert basketball players. Eur. J. Neurosci. 35: 1646–1654. 10.1111/j.1460-9568.2012.08104.x 22541026

[8] Reingold EM, Charness N, Pomplun M, Stampe DM (2001) Visual span in expert chess players: Evidence from eye movements. Psychol. Sci. 12(1): 48–55. 10.1111/1467-9280.00309 11294228

[9] Chase WG, Simon HA (1973) Perception in chess. Cognitive Psychol. 4: 55–81.

[10] Schmidt HG, Boshuizen HP (1993) On the origin of intermediate effects in clinical case recall. Mem Cognition. 21: 338–351. 10.3758/bf03208266 8316096

[11] Reitman JS (1976) Skilled perception in Go: deducing memory structures from inter-response times. Cognitive Psychol. 8: 336–356.

[12] de Groot AD (2008) Thought and choice in chess, AUP.

[13] Charness N (1983) Age, skill, and bridge bidding: A chronometric analysis. J. Verb. Learn. Verb. Beh. 22(4): 406–416.

[14] Ericsson KA, Charness N (1994) Expert performance. Am Psychol. 49(8): 725–747.

[15] Chessgames Services LLC (2001–2012) Chessgames.com: online chess database and Community: Statistics page. Available: http://www.chessgames.com/chessstats.html. Accessed 2012 Jul 9.

[16] Ericsson KA (2006) An introduction to “the Cambridge handbook of expertise and expert performance: Its development, organization, and content”. K.A. Ericsson, N. Charness, P. J. Feltovich, R. R. Hoffman, Eds. Cambridge, MA: CUP, 3–20.

[17] Sutter JD (2012) Wired for success or destruction? Available: http://www.cnn.com/interactive/2012/08/tech/gaming.series/korea.html?hpt=hp_bn5. Accessed 2012 Aug 24.

[18] Blizzard Entertainment (2011) Master League. Available: http://us.battle.net/(developer post) http://us.battle.net/sc2/en/blog/2053471#blog. Accessed 2013 Jan 21.

[19] Pashler H, Johnston JC (1989) Chronometric evidence for central postponement in temporally overlapping tasks. Q J Exp Psychol-A. 41(1): 19–45.

[20] Schneider W, Schiffrin RM (1977) Controlled and automatic human Information processing: I. Detection, search, and attention. Psyc Rev. 84(1): 1–66.

[21] Schiffrin RM, Schneider W (1977) Controlled and automatic human information processing: II. Perceptual learning, automatic attending, and a general theory. Psyc Rev. 84(2): 127–188.

[22] Sellers BC, Fincannon T, Jentsch F (2012) The effects of autonomy and cognitive abilities on workload and supervisory control of unmanned systems. Proceedings of the human factors and ergonomics society 56th annual meeting 56: 1039–1043.

[23] Salvucci DD, Goldberg JH (2000) Identifying Fixations and Saccades in Eye-Tracking Protocols. In Proceedings of the eye tracking research and applications symposium. New York: ACM Press: 71–88.

[24] Land MF, Hayhoe M (2001) In what ways do eye movements contribute to everyday activities? Vision Res. 41: 3559–3565. 10.1016/s0042-6989(01)00102-x 11718795

[25] Breiman L (1984) Classification and regression trees. Boca Raton: Chapman & Hall/CRC. ISBN 0–412–04841–8.

[26] Breiman L (1996) Bagging Predictors. Mach. Learning 24: 123–140.

[27] Breiman L (2001) Random Forests. Mach. Learning 45: 5–32.

[28] Strobl C, Malley J, Tutz G (2009) An introduction to recursive partitioning: rationale, application and characteristics of classification and regression trees, bagging and random forests. Psychol Methods. 14(4): 323–348. 10.1037/a0016973 PMC2927982 19968396

[29] Strobl C, Boulesteix A, Zeileis A, Hothorn T (2006) Bias in random forest variable importance measures: Illustrations, sources and a solution. BMC Bioinformatics 8(25) doi:10.1186/1471-2105-8-25. 10.1186/1471-2105-8-25 PMC1796903 17254353

[30] Linkletter C, Bingham D, Hengartner N, Higdon D, Ye KQ (2006) Variable selection for gaussian process models in computer experiments. Technometrics 48(4): 478–490.

[31] Ericcson AK, Towne TJ (2010) Expertise. Wiley Interdisciplinary Reviews: Cognitive Science. 1(3): 404–416. 10.1002/wcs.47 26271380

[32] Mané A, Donchin E (1989) The Space Fortress game. Acta Psychol. 71: 17–22.

[33] Stern Y, Blumen HM, Rich LW, Richards A, Herzberg G, et al (2011) Space Fortress game training and executive control in older adults: A pilot intervention. Aging Neuropsycholol C 18(6): 653–677. 10.1080/13825585.2011.613450 PMC3591462 21988726

[34] Lewis JM, Trinh P, Kirsh D (2011) A corpus analysis of strategy video game play in StarCraft: Brood War. In expanding the space of cognitive science: Proceedings of the 33^rd^ annual conference of the cognitive science society. L. Carlson, C. Hoelscher, T. F. Shipley, Eds. Austin, TX. 687–692.

[35] Simon HA, Chase WG (1973) Skill in chess: Experiments with chess-playing tasks and computer simulation of skilled performance throw light on some human perceptual and memory processes. AmSci. 61(4): 394–403.

