# Supplementary material for: Video Game Telemetry as a Critical Tool in the Study of Complex Skill Learning
Source: PLoS One. 2013 Sep 18;8(9):e75129. doi: 10.1371/journal.pone.0075129 (PMC3776738; doi:10.1371/journal.pone.0075129)
Supplement: Materials S1 — Supplemental Materials and Methods. (DOC) [file pone.0075129.s017.doc]

**Supplementary Materials**

Table of Contents:

Recruiting and Parsing Replays

Definition of Variables

Definition of Terms

An Overview of the Classifiers

Abilities Coding

Recruiting and Parsing Replays

Participants were StarCraft 2 players who filled out a survey containing the following elements. First, players were asked a series of questions probing StarCraft experience, age, and country. Secondly, players supplied a StarCraft 2 Battle.net identification key, which allowed us to independently confirm the player's league from [www.sc2ranks.com](http://www.sc2ranks.com/), a website tracking statistics for each StarCraft 2 player in a league. Thirdly, players were asked to provide a replay from one of their recent StarCraft 2 games. The replays contain a list of all the commands issued by both players and their opponents in a game that takes five to forty minutes to complete. We set up a website to collect survey data and replay files. These files are encoded in a proprietary format of Blizzard entertainment. We used the free software analysis tool SC2Gears to parse the replay files into text files containing (a) a list of every action in the game, timestamped, performed by either the survey player or their opponent, (b) summary information about the game as a whole, and (c) a log containing all of the online chat the players used to correspond. These text-files were then parsed, uploaded into a MySQL database, and analyzed using scripts developed in MATLAB. Time measurement in the paper was given by the parsed timestamps using the following conversion: 88.5 timestamp units = 1 second. The estimation was made on the basis of the ratio between the max timestamp of each game (leaving the game constitutes an action) and its length as recorded in the game information summary.

Definition of Variables

PAC Variables

Actions in PAC

The mean number of actions within a PAC (1 is the smallest possible value).

Action Latency

Mean Latency (in milliseconds) to first action within a PAC.

Gap between PACs

Mean distance (in milliseconds) between PACs.

Number of PACs per minute

Count of PACs per minute.

Hotkey Variables

Assign to Hotkeys per minute

Players may assign a collection of units to a key from 0 to 9 at any time. The value of this variable refers to number of assignments per minute.

Select by Hotkeys per minute

Once a collection of units has been assigned to a hotkey, players may select those units at any time in order to give them orders. The value of this variable refers to the number of these selections per minute.

Unique Hotkeys

Unique hotkeys refers to the number of keys used for assignment throughout the game.

Complex Unit Production and Use Variables

Complex Ability Use per minute:

The number of ground or unit targeted abilities per game minute (abilities listed as C in our ability coding). These abilities require the player to click, with their mouse, on a specific location or game unit. This is also typically done under time constraints.

Complex Units Made per minute

Number of "ghosts", "infestors", and "high templar" units trained by the player per minute.

Unique Units Made

Number of different unit types (probes, marines, roaches, etc.) produced in game.

Workers Made per minute

The count of workers (a "Probe", "Drone", or "SCV") created by a player per minute. This units production is required yet is relatively independent from the other activities of the player. Its production can therefore be thought of as a dual-task scenario that all players must overcome.

Direct measures of Attentional Control

Minimap Attacks per minute

Number of attack commands that were "off-screen" per minute. This behavior requires attention to a small version of the overall game map. It provides important general information about the current game state.

Minimap right-clicks

Number of right-click ("contextual-key") commands that were "offscreen" (see definition of terms) per minute. This behavior requires attention to a small version of the overall game map. It provides important general information about the current game state.

Total Map Explored

Count of unique Grid (see definition of terms) locations which encompassed at least one screen PoV location per minute

Actions Per Minute (APM)

Count of all actions per minute.

Definition of Terms

Commands/actions

The following were returned by the SC2Gears parser but not considered commands/actions.

Screen movements

Seld

Leaving the game

'Use ability 5f01'

'Use ability 6e00'

'Use ability 7b42'

'Use ability 12940'

'Use ability 14442'

'Use ability 15d09'

'Use ability 17200'

'Use ability 17801'

'Use ability 17901'

'Use ability 1fa0e'

Grid

The game map was divided into a grid of squares 24 x 24 game-coordinates in size.

League

The leagues "Bronze", "Silver", "Gold", "Platinum", "Diamond", "Masters", "GrandMasters", "Professional" were coded 1:8.

Off-screen

Commands to game coordinates that were either (a) farther east or west than 20 game coordinates from screen centre, (b) farther north than 15.5 game coordinates from screen centre, or (c) farther south than 6 coordinates from screen centre. Commands at off-screen locations must be issue by clicking on the mini-map.

Perception Action Cycle (PAC)

PoVs with at least one action

An Overview of the Classifiers

Decision Trees

Before discussing Random Forests, it is helpful to begin by discussing a simpler concept called a Decision Tree. The simplest Decision Tree is a tool used to map many predictor variables (also called explanatory or independent variables) onto a categorical response (when the response is continuous, we use instead a tool called “Regression trees”). We begin by defining a bit of notation. The categorical response is denoted by Y, and the p explanatory variables are denoted by x1, x2… xp. The entire set of explanatory variables is given by X.

A decision tree contains a set of nodes N that contain information about the response given certain combinations of explanatory variables. Specifically, a decision tree makes a prediction about what the response should be given the explanatory variables; in other words, the tree makes a decision about which class it thinks the response should be in. The starting node (also called the root node) is generally depicted at the top of the tree – each prediction starts here and follows a logical path to a terminal node (also called a leaf).

The simplest decision tree is called a binary decision tree: here we use the word binary to mean that each non-leaf node has exactly two children – nodes that can be traveled to from this node. Which of the nodes is traveled to depends on the data point that we are predicting. At each non-leaf node, the tree contains a logical test of the form “is xk greater than Q” for some k and Q. If the logical test yields “true”, then we traverse to the right child, alternatively, if the test yields “false”, we traverse to the left.

An illustration of this is given in Figure S16. Let us see what happens when we try to predict a point with x1=1.5 and x2=-1. We begin at the root node. We are asked whether x1 is more or less than 1. x1 is greater than 1, so we proceed to the left child. We are then asked whether x2 is more or less than 0. Since x2 is less than 0, we proceed to its right child. This tree classifies the data point with x1=1.5 and x2=-1 as Class 2.

Building Decision Trees

The growth of a decision tree starts at the root. The decision tree starts its growth pattern by considering each variable in turn, and considering a split in the form of a logical test considered above, i.e. xk >= Q. For a dataset D with N observations, a continuous variable (assuming no ties) has N-1 possible splits. A categorical explanatory variable with L levels has 2L-1 possible subsets of levels. The tree tries all variables, and for each variable, tries all possible splits. For each split, we evaluate a criterion that tells us how much information is gained by using this split – one simple criterion is the change in misclassification rate. The split that gives the greatest change in misclassification rate is chosen. After splitting, the node constructs two paths: one to a left child and one to a right child. We also split the data D into a set that moves to the left node (all the data that fail the logical test given by the split) and the rest of the data move to the right node. The process then repeats for each child; we note that it is not necessarily the case that the children produce the same split or even split on the same variable.

The decision tree typically has a few stopping criteria. It is standard to never split on nodes with fewer than, say, 5 data points. Furthermore, it is typical to require a minimum decrease in misclassification rate – if no splits provide a sufficient boost in performance, then the tree will not split at that node. This is equivalent to requiring a certain p-value in order to make a split.

Random Forests

A Random Forest is a collection of many Decision Trees. In a Random Forest, instead of having a data point be predicted by a single tree, we now predict it on T trees. The data point now has T classifications: typically we consider them as votes towards a final classification. The class that gets the most votes is considered the classification from the Random Forest.

Building of a Decision Tree is a deterministic process, so it follows that several Decision Trees based off the same data would look identical. Having a forest of identical decision trees would be useless, so it is important to perturb the data in some way. There are two key random features that Random Forests use. The first key randomized feature of the random forests is that the data fed to the Decision Trees is actually a sample (with replacement) of size N from the original data, also known as a bootstrap sample. Each of the T trees in the random forest gets its own sample, so the trees are not based off the same data. The other important randomization is that now, at each node of each Decision tree, we do not consider each variable to split on. Instead, we begin by choosing a random subset of the P predictors (the size of the subset is usually the square root of P for classification, although this can be changed). After choosing a subset of the predictors, we evaluate all possible splits on these and split as usual. The subset can (and does) change within a tree: the subset at the root node is very likely different than the subset at the left child of the root.

Conditional Inference Trees

One of the major problems with using decision trees as a base learner for Random Forests is that they tend to select variables in a biased fashion[29]. Continuous explanatory variables (assuming no ties) have N-1 possible splits, whereas a binary explanatory variable only has 1 possible split. Under the standard decision tree framework, we compare all possible splits at the same time and choose the one that minimizes the misclassification – it should seem apparent here that a continuous variable that gets N-1 “tries” to minimize the criterion gets selected somewhat more than a binary variable with only a single possible split. Conditional Inference Trees were designed to alleviate this problem.

Instead of computing the misclassification under all possible splits, a Conditional Inference tree node first conducts a type of regression on each covariate individually against the response. If the response and explanatory variable are both continuous, the regression is typically linear regression, if the explanatory variable is a discrete factor but the response is continuous, the method is usually ANOVA, and if the response is discrete then a form of a contingency table test is typically used. From each regression we get a p-value, and we compare the p-values from each of the P explanatory variables. We choose the variable with the lowest p-value, and we choose to split on that variable. If the variable has several possible splits, only now do we evaluate every possible split and choose the best one. If none of the variables have a p-value less than 0.05, then we terminate splitting on this sub-tree.

Because this procedure evaluates the variables’ importance on a fairer criterion than “which split is the single best split”, Conditional Inference Trees are the best method of assessing variable importance when the types of variables involved are mixed.

We implemented our analysis using the cforest function in R, from the package party. We use ntree=1000 conditional inference trees in a single random forest (as stated before, we use 25 random forests for each dataset). We use the unbiased control options, as this takes into account the different types of explanatory variables. At each split the classifier considers splitting on mtry= 5 randomly selected explanatory variables in the reported analysis, although we have tried 2, 3, and 4 as well (finding no appreciable difference in overall results).

Variable importance

When provided with only non-predictive variables, a random forest classifier has a baseline classification rate based on the ratio of the sample size of the two groups. For example, if Group A has 55 data points while Group B has 45, and no variable is predictive, the classifier will attempt to achieve maximum classification rate by classifying every data point as a member of Group A. This will result in a baseline correct classification rate of 55%. When the classifier is provided with a predictive variable, the performance of the classifier will, on average, improve.

We measure the so-called "permutation importance" of a single variable by calculating the drop in classification rate when the information in that variable is removed from the dataset (the algorithm accomplishes this by randomly permuting the values of this variable across the rows of the dataset). For every variable, in each of the five classifiers described in Figure 1, we calculated their permutation importance from the classifier. To control for the variability in the Random Forest procedure, we ran each classifier 25 times, each time using a random sample of 70% of the data (without replacement). This produced a distribution of importance values (see Figures 2-7). In each classifier, a vector of noise generated from the standard normal distribution was included as a control variable. Following Linkletter *et al*.[30], a variable which has median performance higher than the 95% percentile of the control is considered an important predictor of league. The classification rate for each classifier is the average out-of-bag prediction accuracy of the 25 runs. Although this method does not give a *p*-value for any variables, it does test the variables with a 1-tailed test at a significance level of α=0.05. Linkletter *et al*.'s method makes no adjustment for multiple comparisons, as it is intended for screening.

Abilities Coding

This is a characterization of the special abilities outputted by SC2Gears. We code them as complex or not complex (Complex=C; Not Complex=N). The list is also included to allow for reproduction of our analysis.

250mm Strike Cannons (Thor) C

Activate/Deactivate Auto-attack Structur N

Activate/Deactivate Auto-repair (MULE) N

Activate/Deactivate Auto-repair (SCV) N

Archon Warp (High Templar or Dark Templa N

Arm Silo with Nuke (Ghost Academy) N

Assault Mode (Viking) N

Attack N

Attack (Bunker) N

Blink (Stalker) C

Burrow (Baneling) N

Burrow (Drone) N

Burrow (Hydralisk) N

Burrow (Infested Terran) N

Burrow (Infestor) N

Burrow (Queen) N

Burrow (Roach) N

Burrow (Ultralisk) N

Burrow (Zergling) N

Calldown Extra Supplies (Orbital Command N

Calldown MULE (Orbital Command) N

Cancel N

Cancel Brood Lord Morphing (Corruptor) N

Cancel Graviton Beam C

Cancel Greater Spire Upgrade (Spire) N

Cancel Hive Upgrade (Lair) N

Cancel Lair Upgrade (Hatchery) N

Cancel Orbital Command Upgrade (Command N

Cancel Overseer Morphing (Overlord) N

Cancel Planetary Fortress Upgrade (Comma N

Cancel Tac Nuclear Strike (Ghost) N

Cancel an Addon (Barracks) N

Cancel an Addon (Factory) N

Cancel an Addon (Starport) N

Chrono Boost (Nexus) N

Cancel Baneling Morphing (Zergling) N

Cloak (Banshee) N

Cloak (Ghost) N

Contaminate (Overseer) C

Corruption (Corruptor) C

Decloak (Banshee) N

Decloak (Ghost) N

EMP Round (Ghost) C

Explode (Baneling) N

Feedback (High Templar) C

Fighter Mode (Viking) N

Force Field (Sentry) C

Fungal Growth (Infestor) C

Gather Resources Protoss (Probe) N

Gather Resources Terran (SCV) N

Generate Creep (Overlord) N

Graviton Beam (Phoenix) C

Guardian Shield (Sentry) N

Halt (on building) N

Halt (on unit) N

Heal (Medivac) N

Hold fire N

Hold position N

Infested Terran (Infestor) C

Land (Barracks) N

Land (Command Center) N

Land (Factory) N

Land (Orbital Command) N

Land (Starport) N

Leave game N

Lift Off (Barracks) N

Lift Off (Command Center) N

Lift Off (Orbital Command) N

Lift Off (Starport) N

Load (Bunker) N

Load (Warp Prism) N

Load All(Command Center/Planetary Fortre N

Lower Supply Depot (Supply Depot) N

Mass Recall (Mothership) C

Morph to Baneling (Zergling) N

Morph to Brood Lord (Corruptor) N

Morph to Overseer (Overlord) N

Move N

Mutate into Greater Spire (Spire) N

Mutate into a Gateway (Warp Gate) N

Neural Parasite (Infestor) C

Patrol N

Phasing Mode (Warp Prism) N

Psionic Storm (High Templar) C

Raiser Supply Depot (Supply Depot) N

Repair (MULE) N

Repair (SCV) N

Research Protoss Blink N

Research Protoss Charge N

Research Protoss Extended Thermal Lance N

Research Protoss Gravitic Booster N

Research Protoss Gravitic Drive N

Research Protoss Graviton Catapult N

Research Protoss Psionic Storm N

Research Protoss Warp Gate N

Research Terran 250mm Strike Cannons N

Research Terran Behemoth Reactor N

Research Terran Caduceus Reactor N

Research Terran Cloaking Field N

Research Terran Combat Shield N

Research Terran Concussive Shells N

Research Terran Corvid Reactor N

Research Terran Durable Materials N

Research Terran Infernal Pre-Igniter N

Research Terran Moebius Reactor N

Research Terran Nitro Packs N

Research Terran Personal Cloaking N

Research Terran Seeker Missile N

Research Terran Siege Tech N

Research Terran Stimpack N

Research Terran Weapon Refit N

Research Zerg Adrenal Glands N

Research Zerg Burrow N

Research Zerg Centrifugal Hooks N

Research Zerg Chitinous Plating N

Research Zerg Glial Reconstitution N

Research Zerg Grooved Snipes N

Research Zerg Metalic Boost N

Research Zerg Neural Parasite N

Research Zerg Pathogen Glands N

Research Zerg Pneumatized Carapace N

Research Zerg Tunneling Claws N

Research Zerg Ventral Sacks N

Return Cargo (Drone) N

Return Cargo (MULE) N

Return Cargo (Probe) N

Return Cargo (SCV) N

Root (Spine Crawler) N

Root (Spore Crawler) N

Salvage (Bunker) N

Scan Move N

Scanner Sweep (Orbital Command) N

Seeker Missile (Raven) C

Set Alliance N

Set Rally Point (Command Center) N

Set Rally Point (Factory) N

Set Rally Point (Hatchery) N

Set Rally Point (Nexus) N

Set Worker Rally Point (Hatchery) N

Set rally point N

Siege Mode (Siege Tank) N

Sniper Round (Ghost) C

Spawn Changeling (Overseer) C

Spawn Creep Tumor (Queen) N

Spawn Larva (Queen) N

Stimpack (Bunker) N

Stimpack (Marauder) N

Stimpack (Mixed Units) N

Stop N

Stop (Bunker) N

Stop Generating Creep (Overlord) N

Tac Nuclear Strike (Ghost) C

Tank Mode (Siege Tank) N

Transfusion (Queen) C

Transport Mode (Warp Prism) N

Unburrow (Baneling) N

Unburrow (Drone) N

Unburrow (Hydralisk) N

Unburrow (Infestor) N

Unburrow (Queen) N

Unburrow (Roach) N

Unburrow (Ultralisk) N

Unburrow (Zergling) N

Unload All (Bunker) N

Unload All (Command Center/Planetary For N

Unload All (Nydus Worm) N

Unload All (Warp Prism) N

Unload All At (Medivac) N

Unload All At (Overlord) N

Unload All At (Warp Prism) N

Unload All (Medivac) N

Unload All (Overlord) N

Unload All (Warp Prism) N

Unload Unit (Bunker) N

Unload Unit (Command Center/Planetary Fo N

Unload Unit (Medivac) N

Unload Unit (Nydus Worm) N

Unload Unit (Overlord) N

Unload Unit (Warp Prism) N

Upgrade Protoss Air Armor 1 N

Upgrade Protoss Air Armor 2 N

Upgrade Protoss Air Armor 3 N

Upgrade Protoss Air Weapons 1 N

Upgrade Protoss Air Weapons 2 N

Upgrade Protoss Air Weapons 3 N

Upgrade Protoss Ground Armor 1 N

Upgrade Protoss Ground Armor 2 N

Upgrade Protoss Ground Armor 3 N

Upgrade Protoss Ground Weapons 1 N

Upgrade Protoss Ground Weapons 2 N

Upgrade Protoss Ground Weapons 3 N

Upgrade Protoss Shield 1 N

Upgrade Protoss Shield 2 N

Upgrade Protoss Shield 3 N

Upgrade Terran Building Armor N

Upgrade Terran Hi-sec Auto Tracking N

Upgrade Terran Infantry Armor 1 N

Upgrade Terran Infantry Armor 2 N

Upgrade Terran Infantry Armor 3 N

Upgrade Terran Infantry Weapons 1 N

Upgrade Terran Infantry Weapons 2 N

Upgrade Terran Infantry Weapons 3 N

Upgrade Terran Neosteel Frame N

Upgrade Terran Ship Plating 1 N

Upgrade Terran Ship Plating 2 N

Upgrade Terran Ship Plating 3 N

Upgrade Terran Ship Weapons 1 N

Upgrade Terran Ship Weapons 2 N

Upgrade Terran Ship Weapons 3 N

Upgrade Terran Vehicle Plating 1 N

Upgrade Terran Vehicle Plating 2 N

Upgrade Terran Vehicle Plating 3 N

Upgrade Terran Vehicle Weapons 1 N

Upgrade Terran Vehicle Weapons 2 N

Upgrade Terran Vehicle Weapons 3 N

Upgrade Zerg Flyer Attacks 1 N

Upgrade Zerg Flyer Attacks 2 N

Upgrade Zerg Flyer Attacks 3 N

Upgrade Zerg Flyer Carapace 1 N

Upgrade Zerg Flyer Carapace 2 N

Upgrade Zerg Flyer Carapace 3 N

Upgrade Zerg Ground Carapace 1 N

Upgrade Zerg Ground Carapace 2 N

Upgrade Zerg Ground Carapace 3 N

Upgrade Zerg Melee Attacks 1 N

Upgrade Zerg Melee Attacks 2 N

Upgrade Zerg Melee Attacks 3 N

Upgrade Zerg Missile Attacks 1 N

Upgrade Zerg Missile Attacks 2 N

Upgrade Zerg Missile Attacks 3 N

Upgrade to Orbital Command (Command Cent N

Upgrade to Planetary Fortress (Command C N

Upgrade to Warp Gate (Gateway) N

Uproot (Spine Crawler) N

Uproot (Spore Crawler) N

Use ability 5f01 N

Use ability 6e00 N

Use ability 7b42 N

Use ability 12940 N

Use ability 14442 N

Use ability 15d09 N

Use ability 17200 N

Use ability 17801 N

Use ability 17901 N

Use ability 1fa0e N

Use ability 30422 N

Vortex (Mothership) C

Yamato Cannon (Battlecruiser) C

Tab N
